# [Expression of Concern] MicroRNA-195 targets VEGFR2 and has a tumor suppressive role in ACHN cells via PI3K/Akt and Raf/ MEK/ERK signaling pathways

**DOI:** 10.3892/ijo.2025.5792

**Published:** 2025-08-20

**Authors:** Pengcheng Sun, Lu Wang, Yunhan Lu, Yuwei Liu, Lechen Li, Luyao Yin, Cheng Zhang, Weiming Zhao, Baozhong Shen, Wanhai Xu

Int J Oncol 49: 1155-1163, 2016; DOI: 10.3892/ijo.2016.3608

Following the publication of the above paper, it was drawn to the Editor's attention by a concerned reader that, regarding the Transwell migration and invasion assay data shown in Fig. 3A and B, two pairs of panels appeared to contain overlapping sections of data (out of a total of ten panels), such that data which were intended to show the results from differently performed experiments appeared to have been derived from the same original sources. Upon analyzing the data independently in the Editorial Office, a third pair of data panels in the same figure were found to be similarly affected.

The authors were contacted by the Editorial Office to offer an explanation for these apparent anomalies in the presentation of the data in this paper, although up to this time, no response from them has been forthcoming. Owing to the fact that the Editorial Office has been made aware of potential issues surrounding the scientific integrity of this paper, we are issuing an Expression of Concern to notify readers of this potential problem while the Editorial Office conitnues to investigate this matter further.

